# SERUM 25-HYDROXYVITAMIN D LEVELS IMPACT RISK OF EXTENSIVE ASSISTANCE NEEDED BY OLDER ADULTS TO WALK

**DOI:** 10.1093/geroni/igz038.1753

**Published:** 2019-11-08

**Authors:** Krista N Jenney, Ronna Robbins, Sara Sweitzer, Nalini Ranjit, Margaret Briley

**Affiliations:** 1 The University of Texas at Austin, Austin, Texas, United States; 2 The University of Texas Health Science Center at Houston School of Public Health in Austin, Austin, Texas, United States

## Abstract

Study objective is to determine the association between deficient 25-hydroxyvitamin D [25(OH)D] serum levels and the amount of assistance needed to walk in a room by older adults living in long-term care (LTC) communities. Participants (age ≥ 65) from five LTC communities in Central Texas were recruited for a multi-site, cross-sectional study (n=169). Double-blinded data abstraction protocols were used to collect a one-year medical history. Laboratory blood draws measured serum 25(OH)D levels. Level of assistance was measured by the activities of daily living score for walking in room from section G of the Minimum Data Set (MDS). To determine the association between deficient 25(OH)D serum levels (≤20 ng/ml) and assistance with walking, adjusted logistic regression was used. Total vitamin D per day (supplementation and meals), therapy and/or restorative programs, body mass index, race, gender, age, and years living in the community were used as confounders. Of the 169 participants (mean age=83) 27.17% had deficient serum 25(OH)D and 9.25% required extensive assistance to walk in a room. The mean serum level and supplementation rate of participants was 32.61 ng/ml and 1,160.64 IU per/d, respectively. Participants with deficient 25(OH)D serum levels had significantly elevated odds (OR=8.73; CL: 1.28, 8.54; p=0.027) of requiring extensive assistance to walk in a room compared to those with adequate serum levels (>20 ng/ml). Deficient 25(OH)D serum levels are associated with increased assistance to walk in a room indicating that adequate serum levels in LTC residents could potentially decrease burden on staff.

